# Proton versus Photon Radiotherapy for Pediatric Central Nervous System Malignancies: A Systematic Review and Meta-Analysis of Dosimetric Comparison Studies

**DOI:** 10.1155/2019/5879723

**Published:** 2019-11-27

**Authors:** Roberta Carbonara, Alessia Di Rito, Angela Monti, Giuseppe Rubini, Angela Sardaro

**Affiliations:** ^1^Interdisciplinary Department of Medicine, Section of Radiology and Radiation Oncology, University of Bari Aldo Moro, Bari, Italy; ^2^Radiation Oncology Unit, IRCCS Istituto Tumori “Giovanni Paolo II”, Bari, Italy; ^3^Interdisciplinary Department of Medicine, Section of Nuclear Medicine, University of Bari Aldo Moro, Bari, Italy

## Abstract

**Background:**

Radiotherapy (RT) plays a fundamental role in the treatment of pediatric central nervous system (CNS) malignancies, but its late sequelae are still a challenging question. Despite developments in modern high-conformal photon techniques and proton beam therapy (PBT) are improving the normal tissues dose-sparing while maintaining satisfactory target coverage, clinical advantages supporting the optimal treatment strategy have to be better evaluated in long-term clinical studies and assessed in further radiobiological analyses. Our analysis aimed to systematically review current knowledge on the dosimetric advantages of PBT in the considered setting, which should be the basis for future specific studies.

**Materials and Methods:**

A PubMed and Google Scholar search was conducted in June 2019 to select dosimetric studies comparing photon versus proton RT for pediatric patients affected by CNS tumors. Then, a systematic review and meta-analysis according to the PRISMA statement was performed. Average and standard deviation values of Conformity Index, Homogeneity Index, and mean and maximum doses to intracranial and extracranial organs at risk (OARs) were specifically evaluated for secondary dosimetric comparisons. The standardized mean differences (SMDs) for target parameters and the mean differences (MDs) for OARs were summarized in forest plots (*P* < 0.05 was considered statistically significant). Publication bias was also assessed by the funnel plot and Egger's regression test.

**Results:**

Among the 88 identified papers, a total of twelve studies were included in the meta-analysis. PBT showed dosimetric advantages in target homogeneity (significant especially in the subgroup comparing PBT and 3D conformal RT), as well as in the dose sparing of almost all analyzed OARs (significantly superior results for brainstem, normal brain, and hippocampal dose constraints and for extracranial OARs parameters, excluding the kidneys). Publication bias was observed for Conformity Index.

**Conclusion:**

Our analysis supports the evidence of dosimetric advantages of PBT over photon RT, especially in the dose sparing of normal growing tissues. Confirmations from wider well-designed studies are required.

## 1. Introduction

Pediatric central nervous system (CNS) malignancies are rare tumors [1] which can arise in different sites of the CNS. In recent years, patients' survival is being increased because of the advances in standard treatments [1, 2]. Radiotherapy (RT) represents a fundamental part of the recommended multimodal therapeutic approaches, even if its late toxicity is still a question of concern in this long-surviving population [3]. In particular, cognitive and endocrine late sequelae are the most common radiation-induced side effects (RISEs) in pediatric patients treated for brain tumors [4]. Furthermore, these children are at increased risk of hearing and visual injuries, as well as vascular diseases and secondary malignant neoplasms (SMNs), depending on the tumor site [4]. Patients treated with craniospinal irradiation (CSI) have reported a decrease in bony growth and damages to extracranial normal organs (such as the lungs and heart) [4].

Technological advances in RT planning and delivery are reducing the exposure of normal tissues, leading to improve toxicity outcomes [5]. Besides continuous advances in image-guided (IG) intensity-modulated (IM) photon radiotherapy, particle therapy with protons is establishing itself as a high-conformal RT modality which is able to improve normal tissues dose sparing while maintaining excellent target coverage [5]. Indeed, thanks to the physical characteristics of protons—such as the typical dose distribution within the “Bragg peak” [6], Proton Beam Therapy (PBT) could represent a safe alternative to photon RT for pediatric tumors or other neoplasms arising next to critical OARs [1]. Nevertheless, radiobiological uncertainties about the interaction of these charged particles with normal and neoplastic cells still persist [6].

A review of dosimetric and toxicity modeling for pediatric medulloblastoma [6] (which compared proton versus photon CSI) confirmed consistent improvements in dose sparing of out-of-field OARs and in reducing the risk of RISEs and SMNs with PBT. Nevertheless, the authors highlighted the lack of evidences from randomized prospective trials and the necessity of appropriate studies with long-term follow-up [6].

Indeed, in the past years, the limited diffusion of PBT centers and the costs of PBT treatments interfered with the account of high-level evidences from large cohorts of patients with long-term follow-up [7]. Nowadays, an increased interest in PBT is supporting its clinical application: ongoing research programs will produce higher-quality data [8].

With the aim to update the knowledge on the dosimetric advantages of PBT in the treatment of pediatric CNS tumors, we performed a systematic review and meta-analysis of published dosimetric studies that compared dosimetric outcomes between PBT and photon RT. The goal is to highlight the main emerging issues in this context that should promote specific researches with the aim to introduce advantages in clinical practice, while supporting clinical data that are being collected.

## 2. Materials and Methods

### 2.1. Search Strategy and Inclusion Criteria

An advanced PubMed search was carried out to answer to the following research question: “What significant advantages for target and OARs dosimetry does PBT provide over photon RT in pediatric treatments for CNS tumors?”

Hence, multiple independent search strategies were performed using the following keywords (in all fields) and arrangements: (Pediatric CNS neoplasms) AND (Proton beam therapy) AND (Radiation therapy) AND (1: Brainstem dose/ 2: Cochlea dose/ 3: Optic chiasm dose/ 4: Hippocampus dose/ 5: Normal brain dose/ 6: Pituitary Gland dose/ 7: Lens dose/ 8: Retina dose/ 9: Lacrimal gland dose/ 10: Circle of Willis dose). To identify studies assessing dosimetric differences for other extracranial OARs in proton versus photon craniospinal irradiation, the keywords (Proton Craniospinal irradiation) AND (Photon Craniospinal irradiation) AND (Dosimetry OR Dosimetric study) were additionally searched.

Searches were completed in June 2019. To identify more references, no restrictions on years or publication type were considered. Indeed, to collect additional eligible studies, we searched supplementary references cited by more recent retrieved review articles. Furthermore, an additional search in Google Scholar was performed for analogous purposes.

A systematic review according to the PRISMA (Preferred Reporting Items for Systematic Reviews and Meta-Analyses) statement [9] was independently conducted by two authors. Study selection criteria—including screening and eligibility requirements—are reported in Table 1. All studies satisfying the eligibility criteria were included in qualitative and quantitative synthesis.

### 2.2. Data Extraction

We collected and analyzed all useful dosimetric data which were provided by the eligible papers for both target volumes and OARs, regardless of the specific search strategies adopted for study selection.

The following basic data were extracted from the included studies: first author name, publication year, tumor histology, sample size, study assessment, and total target dose.

Mean (Dmean) and maximum (Dmax) doses expressed in Gy were specifically considered for our secondary analyses. Whenever possible, it was expected to convert the reported relative values (%) of mean and maximum doses into the corresponding absolute values (Gy). For comparative purposes, average and standard deviation values of Dmean and Dmax were extracted by papers or calculated if raw data were available. If all these data were not available, then the paper was not included in the qualitative synthesis and meta-analysis.

For photon treatments, if the articles provided data by both linac and tomotherapy, we reviewed data of linac-based treatments because of their larger utilization. When both IMRT and VMAT plans were assessed, we extracted and analyzed VMAT data. Similarly, for proton treatments, if the articles provided data by both passively scattered/3D conformal proton therapy and scanning/intensity-modulated (IM) proton therapy, we specifically evaluated data from these latter techniques because of their superior plan quality, as reported in previous published works [1].

### 2.3. Statistical Analysis

We calculated the standardized mean differences (SMDs) with a 95% confidence interval (CI) between photon and proton plans for target dosimetric parameters (Homogeneity Index and Conformity Index). The mean differences (MDs) of Dmax and Dmean values were also calculated between the considered RT modalities with the respective 95% CI.

*I* ^2^ was used to assess heterogeneity between studies. If heterogeneity was not present (*I*^2^ < 50%), a fixed-effect model was performed for our analysis; otherwise, a random-effect model was adopted. *P* < 0.05 was considered statistically significant.

Whenever possible, subgroup analyses were performed to assess differences between photon techniques (3D-CRT versus intensity-modulated techniques).

Publication bias was evaluated by visual inspection of the funnel plot and Egger's regression test. Egger's *P* value <0.1 was considered as significant asymmetry. All statistical analyses were performed using Review Manager (RevMan) version 5.3 and Comprehensive Meta-Analysis version 3.0.

## 3. Results

**Figure 1:**
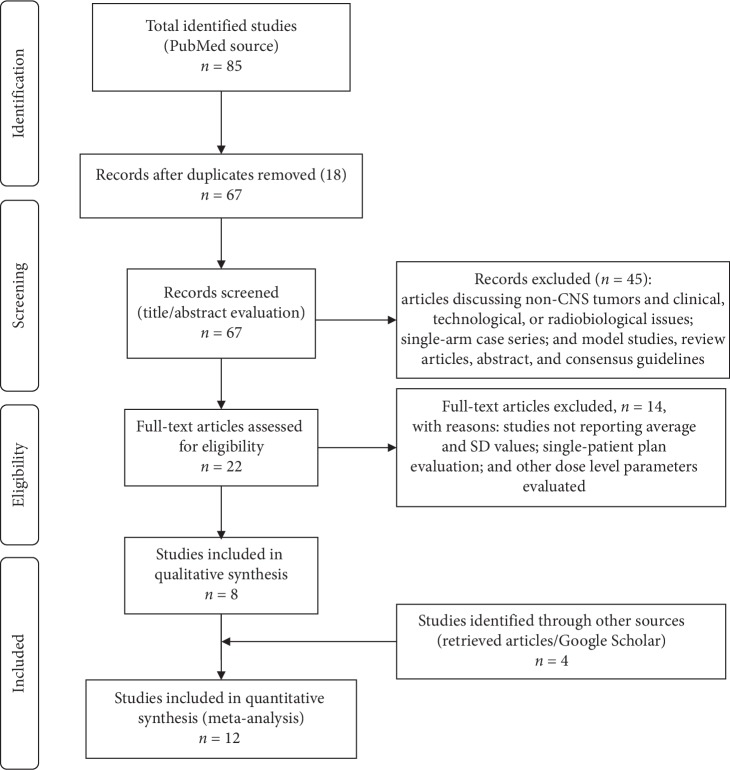
Flow chart of the research strategy according to the PRISMA statement [9].

A total of 88 papers were identified from different sources. PubMed results according to specific keywords were as follows: *brainstem dose n* = 11, *cochlea dose n* = 9, *optic chiasm dose n* = 5, *hippocampus dose n* = 4, *normal brain dose n* = 27, *pituitary gland dose n* = 5, *lens dose n* = 2, and *circle of Willis dose n* = 1; for CSI dosimetric studies, total results *n* = 21. Additional searches on the retrieved articles and Google Scholar provided 4 results.  Figure 1 shows the study flow chart according to the PRISMA statement [9]. Finally, 12 studies were eligible for inclusion in our meta-analysis (Figure 1).

**Figure 2:**
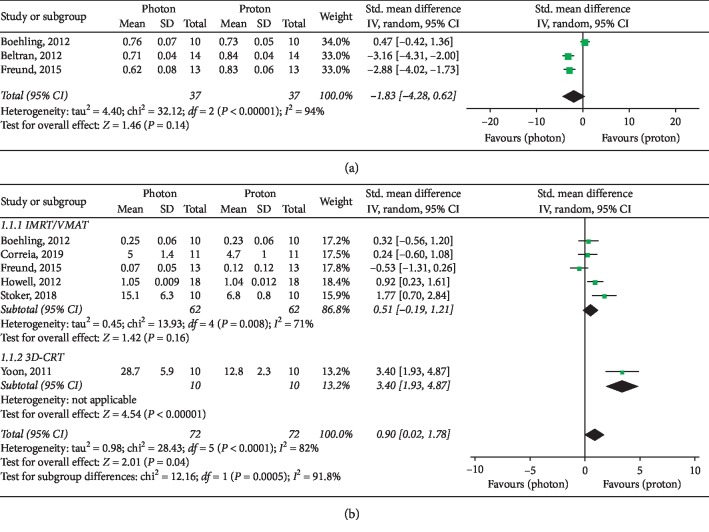
Target conformity and homogeneity. (a) Conformity Index. (b) Homogeneity Index.

**Figure 3:**
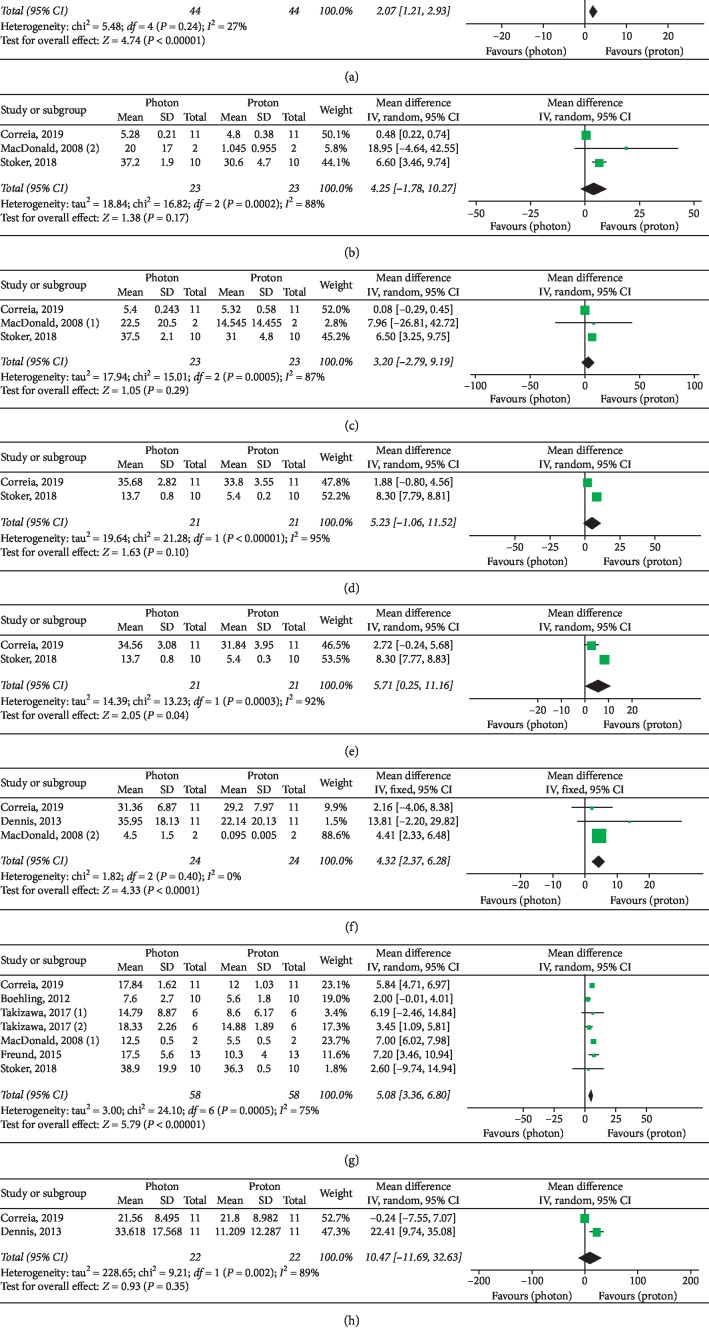
Dmean of intracranial OARs. (a) Brainstem. (b) Left cochlea. (c) Right cochlea. (d) Left hippocampus. (e) Right hippocampus. (f) Optic chiasm. (g) Normal brain. (h) Pituitary gland. When not otherwise specified, the photon RT technique is IMRT or VMAT. Takizawa, 2017 (1): patients affected by ependymoma (IMRT versus PBT); Takizawa, 2017 (2): patients affected by germinoma (IMRT versus PBT); MacDonald, 2008 (1): proton technique: IMPT; MacDonald, 2008 (2): proton technique: 3D conformal PBT.

**Figure 4:**
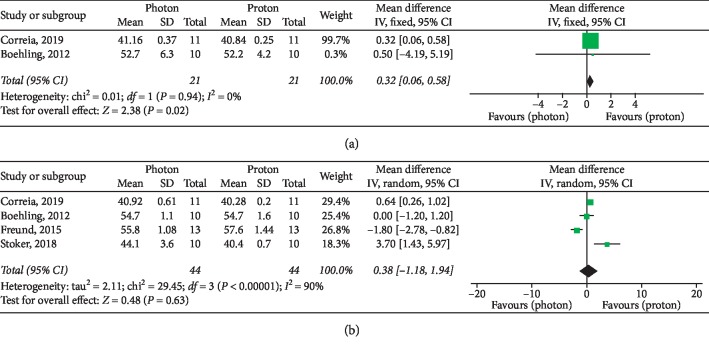
Dmax of intracranial OARs. (a) Brainstem. (b) Normal brain.

**Figure 5:**
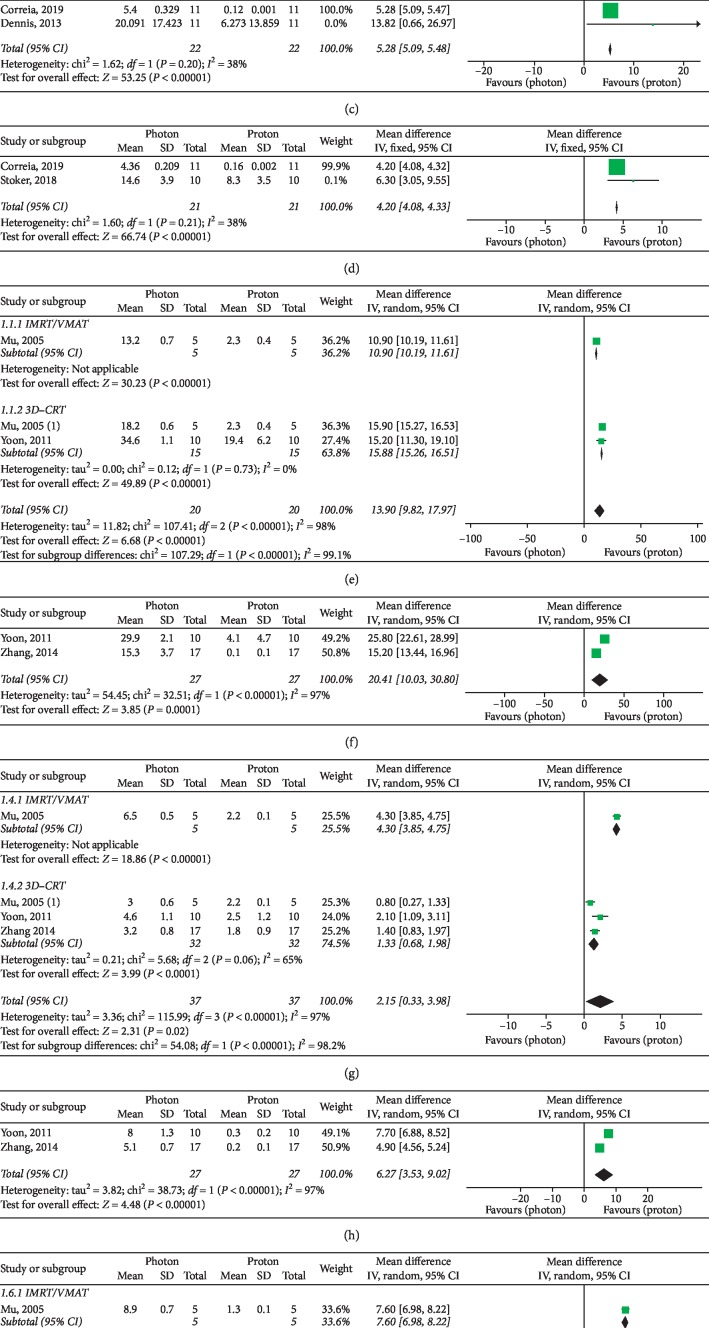
Dmean and Dmax of extracranial OARs. (a) Dmean - left lens. (b) Dmean - left lacrimal gland. (c) Dmean - right lacrimal gland. (d) Dmax - left lens. (e) Dmean - esophagus. (f) Dmean - thyroid. (g) Dmean - lungs. (h) Dmean - liver. (i) Dmean - kidneys. Mu, 2005: IMRT; Mu, 2005 (1): 3D-CRT.

Basic characteristics of included studies are summarized in Table 2. Forest plots for target parameters and OARs doses are shown in Figures 2–5.

Data from single studies—which cannot be aggregated in forest plots for a quantitative synthesis—are summarized in a table submitted as a supplementary material. In case of too small sample size and heterogeneity in the definition of anatomical structures, the results were synthetized in a qualitative manner.

Six studies [10–15]—which included a total of 72 patients—were evaluated for Homogeneity Index assessment. A significant overall advantage in homogeneity of target dose distribution was observed with PBT (SMD: 0.90, 95% CI: 0.02, 1.78, *P*=0.04), with a major improvement in the 3D-CRT subgroup (SMD: 3.40, 95% CI: 1.93, 4.87, *P* < 0.00001) (Figure 2). Nevertheless, no significant differences in the IMRT/VMAT subgroup were observed (*P*=0.16) (Figure 2).

Among the three analyzed studies which provided data for Conformity Index [10, 12, 16], no significant differences were found between the RT modalities (*P*=0.14), even if significant superior results were reported with protons by Beltran et al. [16] and Freund et al. [12] (Figure 2).

Meta-analyses of intracranial OARs mean doses (Figure 3) showed significantly improved results with protons for the following organs: brainstem (MD: 2.07, 95% CI: 1.21, 2.93, *P* < 0.00001), right hippocampus (MD: 5.71, 95% CI: 0.25, 11.16, *P*=0.04), normal brain (MD: 5.08, 95% CI: 3.36, 6.80, *P* < 0.00001), and optic chiasm (MD: 4.32, 95% CI: 2.37, 6.28, *P* < 0.00001). PBT also showed improved results in the studies reporting mean doses to the left and right cochlea, left hippocampus, and pituitary gland, even if these improvements did not provide overall significant differences as compared to photon RT (Figure 3).

Meta-analysis of brainstem maximum doses (Figure 4) revealed a significant advantage with protons (*P*=0.02), while no significant difference emerged for the normal brain maximum dose (*P*=0.63) between the analyzed RT modalities (Figure 4).

Globally, three studies [11, 14, 17] provided useful dosimetric data for extracranial OARs of the cephalic district: mean left lens dose, maximum left lens doses, and mean doses to lacrimal glands were significantly improved in PBT plans (Figure 5). Three supplementary studies [15, 18, 19] assessed dosimetry of cervical, thoracic, and abdominal OARs: mean doses to the esophagus, thyroid, lungs, and liver were significantly improved with PBT, while no significant overall advantage for the kidneys was observed (*P*=0.11) (Figure 5). Nevertheless, subgroup analyses according to photon RT techniques performed for the kidneys showed significant superior results with protons over both intensity-modulated and 3D conformal photon techniques (*P* < 0.00001 in both cases) (Figure 5). The mean difference in the IMRT subgroup was higher than that in the 3D-CRT group: 7.60 (95% CI: 6.98, 8.22) versus 1.47 (95% CI: 1.04, 1.89). A similar higher result in the IMRT subgroup was found for the lungs, as opposed to the results of subgroup analyses performed for the esophagus (Figure 5).

Single studies reporting dosimetric comparisons for the heart mean dose [19], right lens mean dose [14], and optic chiasm and pituitary and lacrimal gland maximum doses [11] substantially confirmed the advantages of PBT over photon RT (see Supplementary Materials) ([Supplementary-material supplementary-material-1]), with the exception of pituitary gland Dmax in Correia's study [11]. Boehling compared mean and maximum doses to vascular structures of the circle of Willis between PBT (with both 3D-PBT and IMPT) and IMRT, confirming the dosimetric advantages of protons [10]. In the study by Correia, only the mean dose to the circle of Willis was reduced by PBT as compared to IMRT and VMAT [11].

### 3.1. Publication Bias

**Figure 6:**
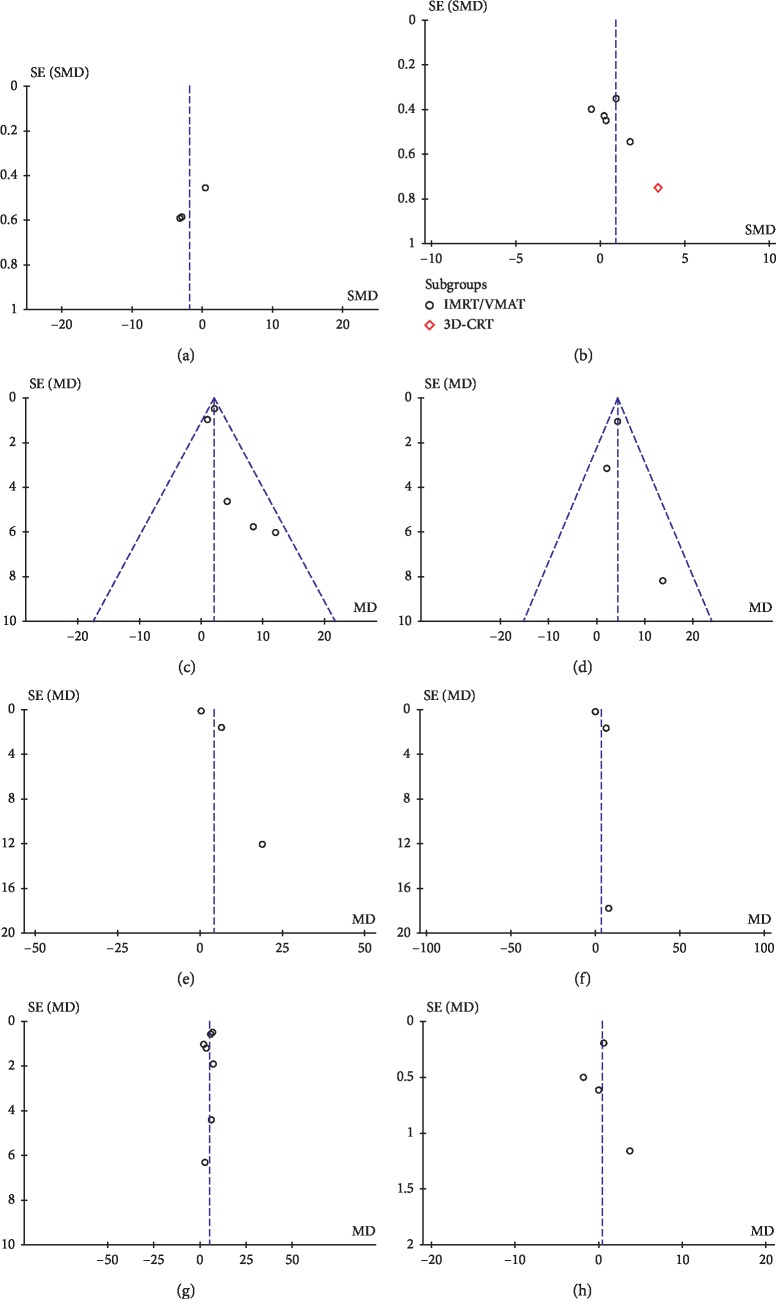
Funnel plots for target and intracranial OARs. (a) Conformity Index. (b) Homogeneity Index. (c) Dmean of the brainstem. (d) Dmean of the optic chiasm. (e) Dmean of the left cochlea. (f) Dmean of the right cochlea. (g) Dmean of the normal brain. (h) Dmax of the normal brain.

**Figure 7:**
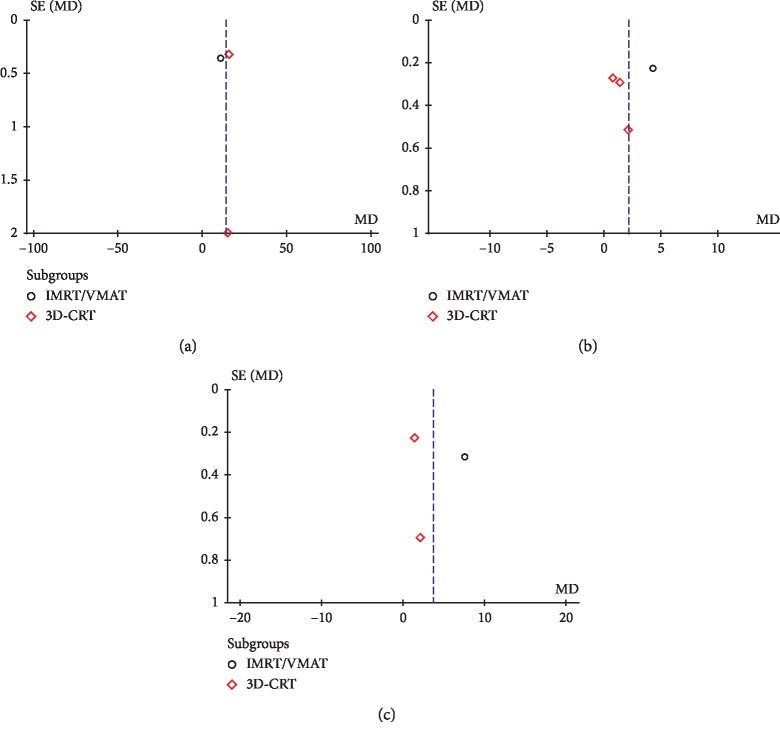
Funnel plots for extracranial OARs. (a) Dmean of the esophagus. (b) Dmean of the lungs. (c) Dmean of the kidneys.

Visual evaluation of funnel plots and Egger's tests were performed for meta-analyses that included at least three studies. Thus, we were able to evaluate publication bias for the following parameters (Figures 6 and 7): target homogeneity and conformity, Dmean of the brainstem, optic chiasm, left and right cochlea, normal brain, esophagus, lungs, and kidneys, and Dmax of the normal brain.

The funnel plot of Homogeneity Index appeared symmetrical, and these findings were confirmed by Egger's regression tests (*P*=0.11), while a significant asymmetry was found for Conformity Index (*P*=0.0088). No significant publication bias was found for all the analyzed OARs (Figures 6 and 7): brainstem (*P*=0.24), optic chiasm (*P*=0.74), left and right cochlea (*P*=0.28 and *P*=0.46, respectively), normal brain (Dmean *P*=0.4, Dmax *P*=0.89), esophagus (*P*=0.99), lungs (*P*=0.61), and kidneys (*P*=0.85).

## 4. Discussion

### 4.1. Radiotherapy in Pediatric CNS Tumors

The 2016 World Health Organization (WHO) classification of CNS tumors [20] emphasizes a huge variety of these neoplasms due to their phenotypical and molecular characteristics which reflect the genetic basis of tumorigenesis.

A variety of tumor histology, grades, and primary locations require different RT prescriptions to provide a radical or adjuvant local disease management. Indeed, different RT treatment fields and doses are used in clinical practice, varying from CSI plus a boost for medulloblastoma/primitive neuroectodermal tumors (PNETs) (depending on the risk level, 24 or 36 Gy could be delivered to the craniospinal axis, followed by a boost to the posterior fossa or to the tumor bed (up to a total dose not inferior to 54 Gy) [4, 21]) and some cases of germ-cell tumors [4, 22] to a resected tumor bed irradiation—e.g., for high-grade glioma, ependymoma (that receives 54 Gy followed by a boost up to 59.4 Gy [4, 22]), craniopharyngioma (prescription doses between 45 and 59.4 Gy have been reported [4, 22]), and some cases of germ-cell tumors [4]—or a whole-ventricular (WV) RT with or without a localized boost (up to 50–54 Gy) for some cases of germ-cell tumors [4, 22].

Previous published authoritative literature [22] has summarized the dosimetric advantages of protons over photons in radiation treatments for pediatric CNS tumors. To our knowledge, to date, this is the first literature review that provides a meta-analysis of dosimetric comparison studies to systematize PBT dosimetric outcomes.

The choice to specifically evaluate data from high-conformal PBT techniques (such as IMPT) when they were available—even if it could have limited the analysis to not very widespread PBT modality—was aimed to improve the knowledge on outcomes of advanced technologies in PBT treatments. The expected findings could support more sophisticated PBT planning and become relevant for medical physicists, medical dosimetrists, and radiation oncologists in the next years.

Similarly, the choice to compare these findings with data from available high-conformal photon techniques was aimed at a preliminary comparative analysis which should encourage further studies, also including cost-effective comparative analyses.

### 4.2. Target Dosimetry Assessment

We compared target dosimetry between proton and photon plans based on the Homogeneity Index and Conformity Index. The first parameter is used to quantify the homogeneity of dose distribution within the target volume, while the second one is used to quantify the conformation of the prescribed dose to the target volume. Superior results in target dosimetry are established according to the highest target conformity (highest value of Conformity Index) and homogeneity (lowest value of Homogeneity Index) [10, 13, 23].

Target conformity and homogeneity were acceptable in all analyzed dosimetric studies. Globally, while homogeneity was significantly improved with PBT—with higher significance in the study by Yoon et al. on CSI [15], confirming the expected advantage of PBT over 3D-CRT [12]—our results showed no significant differences in target conformity and homogeneity between protons and high-conformal photon techniques (IMRT/VMAT) (Figure 2).

The use of high-conformal photon RT has been introduced in clinical practice with the primary aim to improve the dose sparing of normal tissues [23], but these modern techniques substantially improve target conformity and homogeneity, even if major prerequisites for IMRT and VMAT remain the adequate delineation of target volumes and the management of target motion [23]. It is reasonable that the advantages in dose distribution could lead to a safety delivery of higher doses to target volumes, thus improving treatment efficacy.

While we were reaching for useful clinical correlations among the analyzed studies, we observed that MacDonald reported that PBT performed for ependymoma patients was compared favourably with the literature for disease control outcomes [26] over a median follow-up of 26 months.

Confirmations from adequate clinical and radiobiological studies (that should take into account the interaction between protons and tumor cells) are required to clarify the advantage of PBT in tumor control. Results from appropriate cost-effective analyses comparing high-conformal photon RT and PBT will also support the correct management of pediatric CNS tumors.

### 4.3. Dosimetric Analyses for OARs

In our meta-analyses for intracranial and extracranial OARs, we specifically evaluated dosimetric data of OARs involved in neurogenesis (hippocampus [10, 11, 27]), sensory functions (optic chiasm, lens, retina, lacrimal gland, and cochlea), endocrine functions (pituitary gland and thyroid), neurocognition (hippocampus, normal brain, and brainstem), and tissues exposed to SMNs risk (normal brain tissue and brainstem [10, 11]) or late toxicity (circle of Willis for cerebrovascular disease [10, 11] and esophagus, thyroid, heart, lungs, liver, and kidneys).

An adequate respect of intracranial OARs dose constraints (brainstem: Dmax < 54 Gy [27–29]; normal brain: Dmean < 25 Gy [28, 29]; cochlea: Dmean < 35 Gy [27] or <37 Gy [28, 29]; optic chiasm: Dmax < 55 Gy [27] or <52 Gy [28, 29]; hippocampus: Dmean < 30 Gy [27]; and pituitary gland: Dmax < 42 Gy and Dmean < 25 or 30 Gy [27]) was observed in almost all proton and photon plans, but PBT improved the dose sparing for all the analyzed structures (Figures 3 and 4), showing significantly superior results for dose constraints of the brainstem, normal brain, and hippocampus.

Furthermore, even if both proton and photon plans ensured a satisfactory dose sparing for the lacrimal gland (Dmax < 40 Gy [27] and Dmean < 20 Gy [28, 29]), lungs (Dmean < 10 Gy [28, 29]), liver (Dmean < 10 Gy [28, 29]), and kidneys (Dmean < 16 Gy [28, 29]), only PBT achieved a useful dose sparing for the lens (Dmax < 10 Gy [27]), thyroid (Dmean < 6 Gy [28, 29]), and heart (Dmean < 3.5 Gy [28, 29]) among the considered studies. Our analysis also emphasized that PBT provided an improvement in dose sparing of these extracranial OARs, which was significant in all cases except for the kidneys (Figure 5).

In the studies by Boehling et al. and Correia et al. [10, 11], the inappropriate doses to vascular structures of the circle of Willis (which were distinguished by Boehling in anterior and middle cerebral arteries and anterior communicating arteries [10]) were reduced in proton plans. Higher doses to these structures have been shown to correlate with vascular damages, such as Moyamoya syndrome and cerebrovascular disease (ischemic events, in primarily) [10]. Thus, in the case of dose sparing of either vascular structures [10] or other OARs [6], the dosimetric advantages achieved with protons are expected to translate into clinical benefits.

Nevertheless, besides the reduction of OARs doses, Ho et al. [6] underlined the added importance of homogeneous doses to OARs for clinical improvements.

Also, it has to be noted that, since critical structures could be enclosed within the target volume (in-field OARs) or could be in their close proximity when WBRT or WV-RT [11] or CSI [13] is performed, the normal tissues' dosimetric parameters are influenced by the OAR's location [13], as well as treatment fields and total target dose. Accordingly, the degree and extent of neurocognitive deterioration have shown to be affected by the total radiation dose [30] and tumor volume and site, as well as by the age of patient at treatment time [31, 32].

Because of the relevance of these issues in the understanding and disclosing of dosimetric differences between PBT and photon RT for OARs, we underline the heterogeneity of RT treatment fields and target doses among the included studies, which could have influenced our secondary comparisons of plan performance.

In particular, besides the studies on CSI [15, 18, 19], Takizawa et al. [31] and Correia et al. (who also planned a boost to the tumor bed) [11] analyzed whole-ventricular (WV) RT (24–30 Gy) for patients affected by germ-cell tumors, Stoker et al. [14] analyzed hippocampal-avoidance whole-brain RT (total dose: 36 Gy), while other authors analyzed RT to the resected intracranial tumor bed with total target doses in the range between 50.4 and 55.8 Gy (Table 2).

Because of the limited number of included studies, we did not perform subgroup analyses for OARs according to the extent of treatment fields (extended/localized) or total target doses (low/high), but we observed the advantages of PBT in any cases of OAR dose sparing—also including the in-field organs (e.g., brainstem and normal brain) and considering the comparison with modern (high-conformal) photon RT techniques (IMRT/VMAT).

Furthermore, the influence of the OAR's location on absorbed doses can be easily understood: also Howell [13] considered that, during CSI, lungs and kidneys are located bilaterally to the target volume, so they received higher doses in PBT plans as compared to anterior OARs such as the esophagus. This difference observed in spinal treatments is mainly due to the physical properties of the proton beam. Our subgroup analyses according to photon techniques showed a better dose sparing (higher MD) for lateral organs (such as the lungs and kidneys) when PBT is compared to intensity-modulated techniques, and an improved dose sparing of anterior organs (such as the esophagus) when PBT is compared to 3D-CRT. These findings could be related to different beam arrangement between the photon RT techniques.

Globally, the dosimetric benefits for the *in-field*, *partially in-field*, and *out-of-field* OARs obtained with PBT [13]—which are due to the characteristic dose distribution of PBT, dependent on physical properties of protons [13]—could translate into the reduction of neurocognitive damages, visual and hearing loss, endocrine dysfunction, and other late toxicities and SMNs risk. Higher-level evidences from appropriate studies and long-term clinical data are still needed to confirm these suggestions.

With particular regard for SMNs risk assessment, even if we did not specifically analyze risk models, we observed that secondary cancer risks were assessed in some included studies [15, 17–19] that suggested a probabilistic benefit with PBT. Nevertheless, several authors [13, 15, 16] emphasized the concern of neutron contamination risk related to proton treatments in the assessment of SMN risk. On this topic, Beltran, however, observed that a quite low neutron dose for IMPT was reported [16, 33]. More recently, Schneider and Halg [34] have underlined the limitations of previous risk models which assessed the impact of neutron dose. The authors [34] suggested a reduction of SMN risk with PBT when adequate risk models—that take into account well-calculated dose distributions—are used. Globally, it has to be noted that thanks to a reduced integral dose, the risk of SMNs remains lower for PBT as compared to that for photon RT [16, 35], as observed in studies on pediatric CSI that took into account neutron contamination [10, 36] and in a wide retrospective analysis including adult patients [37].

### 4.4. Study Limitations and Additional Considerations

Despite recent advances in radiobiological knowledge, the evaluation of RISEs risk in pediatric patients is still difficult because of the particular radiation sensitivity of developing tissues [1, 38] and the lack of comprehensive radiation dose-volume data in this setting [38]. Indeed, the most used dose constraints for normal tissues reported by QUANTEC (Quantitative Analysis of Normal Tissue Effects in the Clinic) [39] are referred to adults [38] treated with photons. A review of dose constraints and recommendations for intracranial organs at risk (OARs) for both adult and pediatric patients was published in 2015 by Scoccianti et al. [27], even if pediatric constraints were reported in few studies. The choice to consider the Dmean and Dmax values as referring parameters for our secondary analysis was based on an overview of reported normal tissues dose constraints in pediatrics [27–29]. Nevertheless, we are conscious that more informative data are required, such as those which are being expected by collaborative long-term observational studies and ongoing clinical research programs (see the PENTEC (Pediatric Normal Tissue Effects in the Clinic) group project [38]).

The lack of data collected from randomized-controlled trials and the absence of a risk of bias assessment for the individual studies could be considered as limitations of our analysis; nevertheless, we remark that the primary purpose of our work was a secondary analysis of dosimetric comparison studies, which was anyhow feasible because all the included studies analyzed the same patient cohort and compared plans generated with the same treatment planning software.

We however observed a major study limitation in the lack of useful dosimetric data for all the considered OARs: this made us unable to perform a comprehensive secondary analysis for all cases (e.g., when analyzed dosimetric data were reported by single studies) or a complete evaluation of publication bias. Informative data could also have been lost because of specific requirements of our research strategy (e.g., studies reporting the range of average mean doses instead of SD, as well as different dose/volume constraints that were excluded). Also, the limited number and the heterogeneity (heterogeneity can be related to factors such as sex, age, height, and weight that could influence morphometric profiles) of enrolled patients in the included studies can be considered as study limitations.

Furthermore, publication bias was observed for Conformity Index analysis. Indeed, the heterogeneity in the calculation of the considered target dosimetric parameters—which is due to the absence of univocal formulae—could have introduced a potential limitation in our secondary analyses, particularly for Conformity Index assessment.

To reduce the risk of inconsistent results in Conformity Index assessment, we chose to analyze studies [10, 12, 16] that provided a comparison according to analogous formulae [24]. We also agreed with the observation by Ho et al. [6] on the inappropriateness of Conformity Index as the referring parameter for target dose conformation when large target volumes—as those in CSI—are considered. For all these reasons, we excluded Yoon et al. [15] and Howell et al.'s [13] studies from our analysis.

We however considered that a reasonably high level of concordance between different formulae for the calculation of Homogeneity Index has been demonstrated [25]. Additionally, to reduce the potential heterogeneity among studies that assessed target homogeneity, we performed subgroup analyses according to photon RT techniques characterized by different conformation properties. Finally, we remark that we used the standardized mean difference (SMD)—according to the Cochrane recommendations (http://handbook-5-1.cochrane.org)—as a summary statistic to take into account studies that assessed the same outcomes but measured them using a variety of formulae.

Because of the limitations of this meta-analysis, we suggest that the reported results have to be correlated with long-term follow-up data from well-designed studies with larger samples to provide significant information useful in clinical practice. This goal could be more easily achieved, thanks to comprehensive database, as suggested also by Weber et al. [22]. Indeed, the realization of modeling studies for more accurate dose-response and toxicity assessments could benefit from data sharing.

Lastly, we disclose we did not analyze specific dosimetric data for vertebral structures. Only in 2019, a consensus has been published for dose constraints for these structures [40]. Because of the relevance of vertebral bone exposure to children growth, an overview of previously published dosimetric comparison studies—taking into account current dose recommendations—is encouraged to better assess the potential of PBT.

## 5. Conclusions

Our analysis supports current knowledge concerning the dosimetric advantages of PBT over photon RT for pediatric CNS tumors. Protons improve the dose sparing of in-field and out-of-field OARs located in both intracranial and extracranial districts while maintaining satisfactory target conformity and homogeneity. These dosimetric advantages could lead to clinical improvements in pediatric radiation treatments. Wider dosimetric data are necessary to improve the quality of evidence, and further clinical studies and cost-effective analyses comparing photon and proton treatments are required to confirm the benefits of PBT in clinical practice.

## Figures and Tables

**Table 1:** Study selection criteria.

Inclusion criteria	Exclusion criteria
*Screening criteria: population* Pediatric and young adult patients (age < 21 years) affected by CNS neoplasms (craniopharyngioma, ependymoma, neuroblastoma, CNS germinoma, glioma, medulloblastoma, and primitive neuroectodermal tumors (PNETs))	Mixed populations (adults and pediatrics), or adults (age > 21) Non-CNS malignancies

*Screening criteria: study design* Dosimetric comparison between proton beam therapy and photon radiotherapy Dosimetric studies comparing the most advanced/widespread irradiation techniques (protons versus photons)	Studies reporting single techniques and other particle therapy modalities Reviews, clinical case reports/case series, cost-effective studies, simulation studies, preclinical models, etc. Letters, editorials, congress abstracts, and guidelines

*Screening criteria: outcomes* Dosimetric results for target, intracranial OARs (brainstem, cochlea, optic chiasm, hippocampus, normal brain tissue, pituitary gland, and Circle of Willis), and extracranial OARs (lens, retina, lacrimal glands, thyroid, esophagus, lungs, heart, liver, and kidneys)	Absence of reporting of dosimetric outcomes related to the target and/or the considered OARs

*Eligibility criteria: outcome measures* Studies reporting any of the following parameters with average and standard deviation values: (i) For OARs: Dmax for the brainstem, optic chiasm, normal brain tissue, pituitary gland, lens, retina, lacrimal gland, and esophagus and Dmean for the brainstem, cochlea, optic chiasm, hippocampus, normal brain tissue, pituitary gland, circle of Willis, lens, lacrimal gland, and other extracranial OARs (ii) For target: Homogeneity Index and Conformity Index	Studies reporting other dosimetric parameters for target and OARs

*Screening criteria: language* English	All other languages

**Table 2:** Dosimetric studies assessing Conformity Index, Homogeneity Index, and Dmean and/or Dmax for OARs.

Authors (year)	Tumor histology	Patient number	Dosimetric study assessment	Mean total target dose (Gy/RBE/CGE) (dose/fraction)	Evaluation of at least one target parameter: CI (or CN), HI	Dmean/Dmax for OARs (Gy or %) with mean and SD	Conclusions
Stoker et al. (2018) [14]	Primary brain tumors requiring hippocampal-avoidance- (HA-) WBRT	10/20	Dosimetric comparison between VMAT and IMPT for HA-WBRT	36 Gy (1.8 Gy/die) HA-WBRT	HI	Dmax and Dmean reported for the normal brain, hippocampi, cochlea, and lens and Dmean for the brainstem	HA-IMPT can match or improve dosimetric benefits obtained with VMAT.

Freund et al. (2015) [12]	Glioma Ependymoma	8 5	Dosimetric comparison between VMAT, PSPT, and IMPT and risk of cerebral radionecrosis assessment	54 Gy (RBE) (1.8 Gy/die)	CI, HI	Dmax and Dmean evaluated and reported for the normal brain	Both PSPT and IMPT plans significantly improved the maximum dose to the brain. A significant lower risk of brain radionecrosis was observed with PBT.

Howell et al. (2012) [13]	Medulloblastoma	18	Comparison of dose distributions and DVHs between photon and proton CSI	23.4 Gy (1.8 Gy/fr)	CI, HI	Dmean and/or Dmax not reported for the analyzed OARs	Both photon and proton plans provided good target coverage; PBT dose distributions were more homogeneous. Proton CSI improved normal tissue sparing.

Correia et al. (2019) [11]	Intracranial germ-cell tumor	11	Comparison of dose distributions and DVHs between WV-RT/TB IMRT, VMAT, and PBS-PT	24 Gy (RBE) WV-RT plus boost up to 40 Gy (1.6 Gy/fr)	HI and inhomogeneity coefficient	Dmean and Dmax reported (%) for the brainstem, chiasm, normal brain, pituitary gland, circle of Willis, bilateral cochlea, hippocampus, lens, and lacrimal gland	PBS-PT was superior to photons in conformality and OAR sparing.

Boehling et al. (2012) [10]	Craniopharyngioma	10	Comparison of dose distributions and DVHs between IMRT, 3D-PRT, and IMPT	50.4 Gy (CGE) (1.8 Gy/fr)	CN, HI	Dmean and Dmax reported for the vascular OARs, brainstem, and normal brain	PBT was able to avoid excess integral dose to a variety of normal structures at all dose levels while maintaining equal target coverage.
Takizawa et al. (2017) [31]	Ependymoma Germinoma	6 6	Comparison of dose distributions and DVHs between PBT, 3D-CRT, and IMRT	Median of 52.2 Gy for ependymoma and median of 30.6 Gy for germinoma	Not reported	Normal brain dose reported for each patient and as a percentage of the prescription dose (visual inspection of raw data)	PBT reduces the average dose to normal brain tissue as compared to 3D-CRT and IMRT.

MacDonald et al. (2008) [26]	Ependymoma	2/17	Comparison of dose distributions and DVHs between IMPT, 3D-PBT, and IMRT	55.8 Gy	Not reported	Dmean for the brain, brainstem, pituitary gland, optic chiasm, and cochlea evaluated and reported for each patient (Gy)	Dose distributions for PBT were compared favourably with IMRT plans. IMPT allows further sparing of some critical structures.

Beltran et al. (2012) [16]	Craniopharyngioma	14	Dosimetric comparison between IMRT, double-scatter (DS) PT, and IMPT	54 Gy (1.8 Gy/die)	CI	Not reported (other dosimetric parameters are reported)	PBT significantly reduced the dose to the whole brain. IMPT was the most conformal treatment that improved OAR dose sparing, but it was highly sensitive to target changes.

Dennis et al. (2013) [17]	Low-grade glioma	11	Dosimetric (DVH) comparison between IMRT and PBT. SMN risk assessment	54 Gy (1.8 Gy/die)	Not reported	Dmean for the brainstem, pituitary gland, optic chiasm, and lacrimal gland evaluated and reported for each patient (Gy)	PBT improved the reduction of doses to normal tissues, especially when tumors were in close proximity to critical structures. IMRT had a twofold higher risk of SMNs as compared to PBT.

Mu et al. (2005) [18]	Medulloblastoma	5	Dosimetric comparison between conventional photons, IMRT, electrons, and PBT. SMN risk assessment	23.4 Gy (1.8 Gy/die)	Not reported	Dmean evaluated and reported for the thyroid, esophagus, heart, lungs, and liver	IMPT significantly reduced mean doses to OARs, except for the lungs (not significantly). IMPT reduced SMN risk.
Zhang et al. (2014) [19]	Medulloblastoma	17	Dosimetric comparison between PSPT CSI and field-in-field photon CSI. SMN risk assessment	23.4 Gy (1.8 Gy/die)	Not reported	Dmean evaluated and reported for the thyroid, heart, lungs, and liver	PSPT CSI provided lower doses to OARs, superior predicted outcomes, and lower predicted risks of SMNs and cardiac mortality than field-in-field photon CSI.

Yoon et al. (2011) [15]	Various CNS tumors	10	Comparison of dose distributions, DVHs, and SMN risk between CSI with 3D-CRT, TOMO, and PBT. SMN risk assessment	36 Gy (1.8 Gy/fr) to the spine; total target dose ranged between 54 and 60.6 Gy	CI, HI	Dmean evaluated and reported for the lens, thyroid, esophagus, lungs, liver, and kidneys	PBT provided the best HI and a superior CI than 3D-CRT (no significant difference compared to TOMO). OAR doses with PBT were lower than those obtained with 3D-CRT or TOMO. Lower SMN risk was reported with PBT.

CI: Conformity Index; HI: Homogeneity Index; CGE: cobalt Gy equivalents; RBE: relative biological effectiveness; SD: standard deviation; CSI: craniospinal irradiation; TOMO: tomotherapy; PBS-PT: pencil beam scanning-proton therapy; PSPT: passively scattered PT; VMAT: volumetric modulated arc therapy; IMRT/IMPT: intensity-modulated radiotherapy or PT; SMNs: secondary malignant neoplasms; WBRT: whole-brain RT; WV-RT/TB: whole-ventricular RT followed by a boost to the tumor bed.

## Supplementary Materials

Supplementary Materials Table: results of single studies reporting dosimetric comparisons between photon RT and PBT for intracranial and extracranial OARs.
