# Supplementary material for: Proton versus Photon Radiotherapy for Pediatric Central Nervous System Malignancies: A Systematic Review and Meta-Analysis of Dosimetric Comparison Studies
Source: J Oncol. 2019 Nov 27;2019:5879723. doi: 10.1155/2019/5879723 (PMC6900940; doi:10.1155/2019/5879723)
Supplement: Supplementary Materials — Table: results of single studies reporting dosimetric comparisons between photon RT and PBT for intracranial and extracranial OARs. [file 5879723.f1.docx]

Table - Supplementary Materials. Results of single studies reporting dosimetric comparisons between photon RT and PBT for intracranial and extracranial OARs

| **Author,**  **year** | **Photon technique** | **Proton technique** | **N.**  **pt** | **Mean and SD**  **Photon RT** | | **Mean and SD**  **Proton RT** | | **Dose (Gy)** |
| --- | --- | --- | --- | --- | --- | --- | --- | --- |
|  |  |  |  | **Dmax pituitary gland** | | **Dmax pituitary gland** | |  |
| Correia 2019 | VMAT | PBS-PT | 11 | 24,32 | 9,09 | **27,56** | 8,95 | 40 |
|  |  |  |  | **Dmax**  **Optic chiasm** | | **Dmax**  **Optic chiasm** | |  |
| Correia 2019 | VMAT | PBS-PT | 11 | 36,72 | 5,03 | 36,48 | 5,87 | 40 |
|  |  |  |  | **Dmax lacrimal**  **gland, left** | | **Dmax lacrimal gland, left** | |  |
| Correia 2019 | VMAT | PBS-PT | 11 | 7,4 | 0,51 | 2,64 | 0,45 | 40 |
|  |  |  |  | **Dmax lacrimal**  **gland, right** | | **Dmax lacrimal gland, right** | |  |
| Correia 2019 | VMAT | PBS-PT | 11 | 7,08 | 0,45 | 0,52 | 0,02 | 40 |
|  |  |  |  | **Dmean lens, right** | | **Dmean lens, right** | |  |
| Stoker 2018 | VMAT | IMPT | 10 | 13,8 | 5,2 | 5,7 | 3,3 | 36 |
|  |  |  |  | **Dmax lens, right** | | **Dmax lens, right** | |  |
| Stoker 2018 | VMAT | IMPT | 10 | 15,5 | 5,8 | 8,2 | 4 | 36 |
|  |  |  |  | **Dmean heart** | | **Dmean heart** | |  |
| Zhang 2014 | 3D-CRT | PBT | 17 | 10,4 | 2,2 | 0,2 | 0,2 | 23,4 |
| Mu 2005 | 3D-CRT | IMPT | 5 | 11,9 | 0,9 | 0 | 0 **(not applicable)** | 23,4 |
| Mu 2005 | IMRT | IMPT | 5 | 4,8 | 0,3 | 0 | 0 **(not applicable)** | 23,4 |
